# Pancreatic rest disguised as inflammatory bowel disease in young adult

**DOI:** 10.1093/jscr/rjac153

**Published:** 2022-04-11

**Authors:** Karleigh Curfman, Megan Linders, Ashwini Poola, Larry O’Bryant, Laila Rashidi

**Affiliations:** Department of Surgery, Tacoma General Hospital, MultiCare Health System, Tacoma, WA, USA; Department of Surgery, Tacoma General Hospital, MultiCare Health System, Tacoma, WA, USA; Department of Surgery, Tacoma General Hospital, MultiCare Health System, Tacoma, WA, USA; Department of Pathology, Tacoma General Hospital, MultiCare Health System, Tacoma, WA, USA; Department of Surgery, Tacoma General Hospital, MultiCare Health System, Tacoma, WA, USA

**Keywords:** pancreatic rest, ectopic pancreas, aberrant pancreas, inflammatory bowel disease

## Abstract

Pancreatic rest, otherwise known as aberrant, ectopic or heterotopic pancreas, occurs when the pancreatic tissue does not have an anatomical or vascular connection to the normal body of the pancreas. This rare congenital anomaly was first described in 1727 by Hunt and Bonesteel, and it is now known to be found predominantly within the stomach or proximal small bowel. Most of the time, pancreatic rest is asymptomatic and is found incidentally. When symptomatic, the most common presentations tend to be: abdominal pain, nausea, gastrointestinal bleeding, obstruction and symptoms of pancreatitis. We report a case of a 21-year-old female with symptomatic pancreatic rest noted in two locations: antrum of the stomach and the proximal jejunum just distal to the ligament of Treitz.

## INTRODUCTION

Pancreatic rest, or aberrant, ectopic or heterotopic pancreas, is a rare congenital anomaly [1]. The incidence is 0.5–13% from autopsies [1]. These masses are often found incidentally and asymptomatic [1]. When symptomatic, pancreatic rest may cause pain, nausea, bleeding, obstruction and pancreatitis [2]. Masses are typically located in the stomach, duodenum and jejunum [1].

We report a 21-year-old with a 10-year history of pain, nausea and diarrhea, initially treated with regimens aimed toward Crohn’s disease. Despite maximal medical management, symptoms persisted and further evaluation revealed a 2.6-cm jejunal mass. Due to the size, suspected wall involvement and location, biopsy was not feasible and the patient was referred for surgery. Resection was performed utilizing a robotic approach. Upon pathological examination of the specimen, typical pancreatic architecture was present confirming pancreatic rest. The patient is now symptom-free and off medications.

## CASE REPORT

A 21-year-old presented with ongoing pain, nausea and diarrhea. She had a 10-year history of abdominal pain for which she was initially diagnosed with Crohn’s disease at 9 years of age, and later, she felt to have ulcerative colitis as a teenager. Her routine management consisted of mesalamine with burst dose steroids for acute flares. She underwent repeat comprehensive evaluation to assess her persistent symptoms. The patient endured multiple imaging studies including: abdominal ultrasounds, hepatobiliary iminodiacetic acid scan, computed tomography (CT), CT enterography, magnetic resonance imaging (MRI) and MRI enterography. On MRI enterography, a 1.9-cm non-obstructing jejunal lesion was identified. Push enteroscopy was performed revealing submucosal masses in the antrum and jejunum with central umbilication. Biopsies were taken and the jejunal mass tattooed, but pathology was inconclusive.

**Figure 1:**
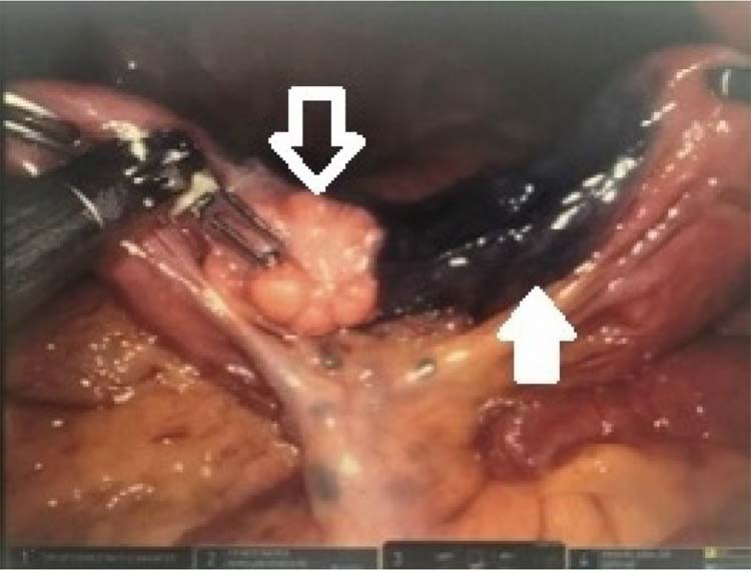
Robotic intraoperative view of identified lesion; the intraabdominal lesion was easily identified (white outlined arrow) immediately adjacent to the previously placed endoscopic tattoo (solid white arrow).

**Figure 2:**
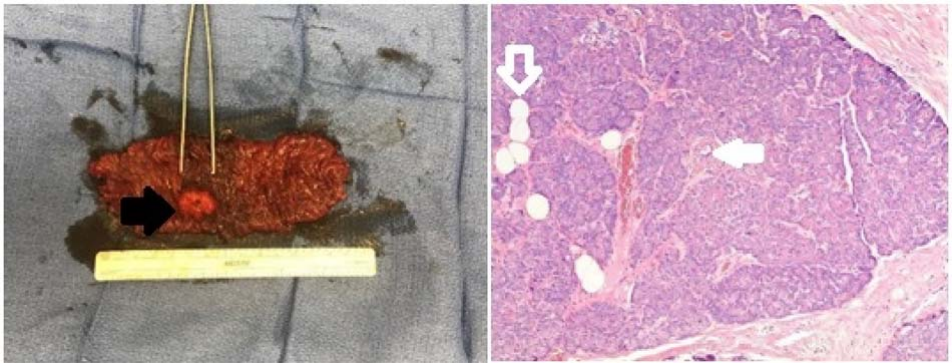
Gross and pathologic representation of resected pancreatic rest; the resected portion of jejunum was opened to reveal an intraluminal mass (solid black arrow); the mass underwent further pathologic evaluation, which revealed acini, ducts (outlined white arrow) and Islet cells (solid white arrow) specific to the pancreas and confirming the diagnosis of pancreatic rest.

Endoscopic ultrasound (EUS) revealed a 1-cm submucosal antral mass. Due to size and wall involvement, pancreatic rest was suspected, though the jejunal lesion multifocal carcinoid tumor remained in the differential. An endoscopic mucosal resection of gastric lesion was performed, but pathology again was inconclusive. As the jejunal mass was noted to be 20–30 cm distal to the ligament of Treitz, and a miniprobe EUS was utilized to access this location. The small bowel lesion was hypoechoic, well demarcated, 2 cm and arose from the muscularis propria. Due to the size, location and depth of wall involvement, an endoscopic biopsy or resection was not feasible and formal surgical resection was recommended. Therefore, a robotic small bowel resection was performed. The previously marked jejunum was identified adjacent to a large mass with associated enlarged mesenteric lymph nodes (Fig. 1). Approximately, 13 cm of jejunum and mesentery were resected (Fig. 2). Pathologic examination revealed a 2.6-cm submucosal mass with pancreatic features, including acini, ducts and Islet cells, confirming pancreatic rest (Fig. 2).

On post-operative outpatient evaluation, she reported complete resolution of all suspected Crohn’s disease symptoms. Specifically, in addition to pain resolution, she reported having normal bowel movements for the first time in 10 years.

## DISCUSSION

Pancreatic rest, aberrant, ectopic or heterotopic pancreas, is a rare congenital anomaly of pancreatic tissue, which occurs in the absence of normal anatomical or vascular connection to the pancreas [1]. It was first described in 1727 by Hunt and Bonesteel within an ileal diverticulum [1]. The incidence of pancreatic rest ranges from 0.5 to 13% in autopsy studies [1]. The majority of heterotopic pancreas are found in the stomach (24–38%), duodenum (9–36%) and jejunum (0.5–27%), though it has been found in the biliary tract, liver, spleen, mesentery, skin, lymph nodes, Meckel’s diverticulum and fallopian tubes [3].

Heterotopic pancreas was categorized by Heinrich in 1909 and was modified by Gaspar-Fuentes in 1973 [3]. Type I consists of tissue most resembling the pancreas containing acini, ducts and Islet cells. Type II contains only pancreatic ducts. Type III resembles exocrine pancreas by containing acinar tissue. Type IV resembles endocrine pancreas as it contains only Islet cells [3]. It is uncertain how exactly pancreatic rest forms, however, embryologic theories have been proposed [3]. The first states that evaginations of the primitive duodenal wall, which form the pancreas, can remain within the bowel and migrate away from the developing organ creating ectopic pancreas [3]. The second theory states that metaplastic pancreatic endodermal tissue settles in various submucosas [3].

Pancreatic rest generally is asymptomatic and most commonly found incidentally on endoscopy, laparotomy or autopsy [1, 2]. Though often nonspecific when symptomatic, frequent symptoms include pain, epigastric discomfort, nausea and vomiting [1–3]. Symptoms depend upon pancreatic rest location and size, where rare cases have presented with obstructive jaundice, gastric/bowel obstruction or hemorrhage [1–3]. Symptoms are typically seen when the lesion is >1.5 cm [3]. Pediatric cases have also shown intussusception and pancreatitis [4]. Several reported cases have also demonstrated abscess and pseudocyst formation [1–3]. Fifteen cases have reported malignant transformation [3]. It is theorized that pain is caused by endocrine and exocrine secretions causing inflammation and chemical irritation of the involved tissue [3]. Additionally dyspepsia, ulcer formation and hemorrhage can be caused by mucosal erosions from enzyme release and intestinal or biliary symptoms from luminal obstruction by ectopic tissue [1, 3].

While CT still poses diagnostic challenges, there are findings that are helpful in making the diagnosis. These include flat ovoid shape, antrum, pylorus, or duodenum location, long:short diameter ratio > 1.4, endoluminal growth with ill-defined borders and enhancement of overlying mucosa [2]. It has been demonstrated that having at least two of these findings had a sensitivity of 100% and specificity of 82.5% in upper gastrointestinal pancreatic rest diagnosis [2]. Additionally, masses have been found to range from 0.2 to 4.0 cm [1]. Upon endoscopic visualization, pancreatic rest usually presents as a broad-based submucosal nodule with umbilication, as seen herein [3]. Endosonographic studies often reveal a hypoechoic, heterogenous lesion arising from the third or fourth layer of the gastrointestinal tract with indistinct margins [4]. Biopsy, frozen section or surgical resection can be used to confirm the diagnosis [1–3].

Overall, pancreatic rest is rare and difficult to diagnose. These challenges were highlighted herein. Diagnostic difficulties are related to nonspecific symptoms, inconclusive testing due to mass location, vague CT findings and difficult biopsy. While pancreatic rest is rare and can be elusive, it should be considered in the differential diagnoses of gastric and small bowel masses.

## AUTHORS’ CONTRIBUTIONS

K.C., M.L. and A.P. were responsible for the conception and design, acquisition of data, interpretation of data and manuscript writing and drafting. K.C., M.L., A.P., L.O’B. and L.R. revised the manuscript critically for important intellectual content and were in charge of the final approval of the version to be published.

## AUTHORS’ DECLARATION

The authors of this report verify that the manuscript has been read and approved by all authors, requirements for authorship have been met and believe that the manuscript represents honest work. An abstract of this report has been previously presented at the Northwest Society of Colon and Rectal Surgeons (NWSCRS) on 24 July 2021. It otherwise has not been published or presented elsewhere.

## DISCLOSURE

An abstract of this report has been previously presented at the Northwest Society of Colon and Rectal Surgeons on July 24, 2021. It otherwise has not been published or presented elsewhere.
